# Autophagy Inhibitor LRPPRC Suppresses Mitophagy through Interaction with Mitophagy Initiator Parkin

**DOI:** 10.1371/journal.pone.0094903

**Published:** 2014-04-10

**Authors:** Jing Zou, Fei Yue, Wenjiao Li, Kun Song, Xianhan Jiang, Jinglin Yi, Leyuan Liu

**Affiliations:** 1 Center for Cancer and Stem Cell Biology, Institute of Biosciences and Technology, Texas A&M Health Science Center, Houston, Texas, United States of America; 2 Jiangxi Research Institute of Ophthalmology and Visual Sciences, The Affiliated Eye Hospital of Nanchang University, Nanchang, Jiangxi, China; Georgia Regents University, United States of America

## Abstract

Autophagy plays an important role in tumorigenesis. Mitochondrion-associated protein LRPPRC interacts with MAP1S that interacts with LC3 and bridges autophagy components with microtubules and mitochondria to affect autophagy flux. Dysfunction of LRPPRC and MAP1S is associated with poor survival of ovarian cancer patients. Furthermore, elevated levels of LRPPRC predict shorter overall survival in patients with prostate adenocarcinomas or gastric cancer. To understand the role of LRPPRC in tumor development, previously we reported that LRPPRC forms a ternary complex with Beclin 1 and Bcl-2 to inhibit autophagy. Here we further show that LRPPRC maintains the stability of Parkin that mono-ubiquitinates Bcl-2 to increase Bcl-2 stability to inhibit autophagy. Under mitophagy stress, Parkin translocates to mitochondria to cause rupture of outer mitochondrial membrane and bind with exposed LRPPRC. Consequently, LRPPRC and Parkin help mitochondria being engulfed in autophagosomes to be degraded. In cells under long-term mitophagy stress, both LRPPRC and Parkin become depleted coincident with disappearance of mitochondria and final autophagy inactivation due to depletion of ATG5-ATG12 conjugates. LRPPRC functions as a checkpoint protein that prevents mitochondria from autophagy degradation and impact tumorigenesis.

## Introduction

Autophagy, or self-digestion, is a process that begins with the formation of isolation membranes that engulf substrates including dysfunctional organelles, mis-folded/aggregated proteins and/or other macromolecules to form autophagosomes [1], [2]. Then autophagosomes fuse with lysosomes to generate autolysosomes in which substrates are degraded [3]. Mitochondrion is one of the most prominent and vital type of organelles in eukaryotic cells. During cell cycling, mitochondria are constantly synthesized, used, damaged and destroyed through autophagy (here referred to as mitophagy) [4], [5]. Parkin, whose mutations may be counted for Parkinson's disease in small numbers of patients, has recently been found to regulate the turnover of mitochondria through mitophagy [6], [7]. The role of autophagy in cancer development has attracted great attention but is not well understood [8].

LRPPRC is an interactive protein of MAP1S, a mitochondria and microtubule-associated protein previously named as C19ORF5 [9]–[11]. It was suggested that mutations in the LRPPRC gene cause Leigh syndrome, French-Canadian type (LSFC), a human disorder characterized with neurodegeneration and cytochrome c oxidase deficiency [12]. Based on the somatic mutation data of 17301 genes from 316 ovarian cancer patients from *The Cancer Genome Atlas*, mutations in both LRPPRC and MAP1S were found to reduce the survival of patients [13]. As a sequence homologue of the microtubule-associated protein MAP1A and MAP1B, MAP1S similarly interacts with mammalian autophagy marker LC3 [14]–[16] and bridges autophagic components with microtubules and mitochondria to affect autophagosomal biogenesis and degradation and suppress genome instability and tumorigenesis [16]–[18]. Recently, we found that elevated levels of LRPPRC in prostate adenocarcinomas are closely associated with poor prognosis of prostate cancer patients [19]. A similar trend was independently reported in patients with gastric cancer [20].

To better understand the role of LRPPRC in cancer development, we have previously reported that LRPPRC associates with mitochondria, interacts with Beclin 1 and Bcl-2 and form a ternary complex to maintain the Bcl-2 stability. Suppression of LRPPRC leads to Bcl-2 degradation that leads to release of more Beclin 1 to form complexes with PI3KCIII to activate basal levels of autophagy upstream of the ATG5-ATG12 conjugates-mediated LC3-I to LC3-II conversion [21]. Since inner mitochondrial membrane-associated LRPPRC [22] was suggested to interact with mitophagy initiator Parkin based on Mass Spectrometry analyses from different labs [23], [24] and its suppression led to enhancement of autophagy degradation of mitochondria in lysosomes [21], we are triggered to investigate the specific role of LRPPRC in mitophagy in addition to its general role in the regulation of basal levels of autophagy.

In this study, we show that LRPPRC interacts with Parkin and maintains the stability of Parkin that stabilizes Bcl-2 to suppress autophagy from initiation. Under mitophagy stress, mitophagy initiator Parkin translocates to depolarized mitochondria to bind with LRPPRC. Consequently, LRPPRC and Parkin regulate VADC1, Drp1 and Mitofusin 1 to initiate autophagy and mitophagy, and eventually become depleted along their associated mitochondria in cells under long-term mitophagy stress. Therefore, LRPPRC protects mitochondria from autophagy degradation.

## Materials and Methods

### Antibodies, siRNAs, plasmids, and other reagents

Antibody against LRPPRC (1B8) was a gift from Dr. Serafín Piñol-Roma, The Sophie Davis School of Biomedical Education, the City College of New York, New York [22]. Antibodies against human LC3 (NB 100-2331) were purchased from Novus Biologicals and A&G Pharmaceutical, Inc.. The IgG control antibodies from mouse (SC-2025) and rabbit (SC-2027), primary antibodies against *β-actin* (SC-47778), LRPPRC (mouse, SC166178; rabbit, SC-66845), ATG5 (SC-33210), Bcl-2 (SC-7832), p27 (SC-776), Beclin 1 (SC-11427), Mitofusin 1 (SC-166644), Drp1 (SC-32898), VDAC1 (SC-98708), and GFP (SC-8334), and siRNA molecules specific to LRPPRC (sc-44734), Parkin (SC-42158) and random sequence control (sc-44234) were from Santa Cruz Biotechnology, Inc.. Antibodies against Parkin (mouse, ab77924; rabbit, ab15954), Pink1 (ab23707) and LAMP2 (ab18528) were from abcam. Antibody against P62 (SQSTN1, BWL-PW9860) was from Enzo Life Sciences International Inc. Antibody against Tom20 (612278) was from BD Transduction Laboratories. HRP-conjugated secondary antibodies against mouse (#172-1011) and rabbit (#172-1019) were from Bio-Rad. Rhodamine Red-X goat anti-mouse IgG and Alexa Fluor 633 goat anti-rabbit IgG (R6393 and A-21070), MitoTracker Red CMXRos, Lipofectamine 2000 (11668-027) and Oligofectamine (12252-011) were from Invitrogen. GFP-LC3 [25] and GFP-Parkin [26] were supplied by Drs. Mizushima and Youle, respectively. MG-132, Bafilomycin A1, Carbonyl cyanide *m*-chlorophenyl hydrazone (CCCP) and 6-hydroxydopamine (6-OHDA) were from Sigma. The protein G beads were from Amersham Biosciences.

### Cell transfection and co-immunoprecipitation assay

HeLa cells or HeLa cells stably expressing EGFP-LC3 that was established as previously described [4], [25] were transfected with siRNA molecules packed with Oligofectamine and/or GFP-Parkin plasmid [25] packed in Lipofectamine 2000 as we previously described [4], [27]. In order to suppress LRPPRC and overexpress GFP or GFP-Parkin simultaneously, cell densities were increased to 50–60% confluence to reduce transfection-induced cell death. Cell lysates were prepared from attached cells and coimmunoprecipitation were performed as previously described [28]. Same amount of cell lysates were subjected to immunoprecipitation with equal amounts of specific antibodies and control antibodies (IgG) from the same species.

### Fluorescent microscopy and transmission electron microscopy

Immunofluorescent stain and mitochondria tracking were performed and images were captured with the laser scanning microscope similarly as described [4], [25]. HeLa cells grown in 6-well culture plates were transfected with LRPPRC-specific siRNA or treated with CCCP in the absence or presence of lysosomal inhibitor Bafilomycin A1, and then fixed and processed for examination with a JEM 1010 transmission electron microscope (JEOL, USA, Inc.) as described [16]. Percentages of areas occupied by autophagic vacuoles or mitochondria were measured using the ImageJ program.

## Results

### Long-term mitophagy stress leads to depletion of LRPPRC and mitochondria and impairment of autophagy/mitophagy flux

**Figure 1:**
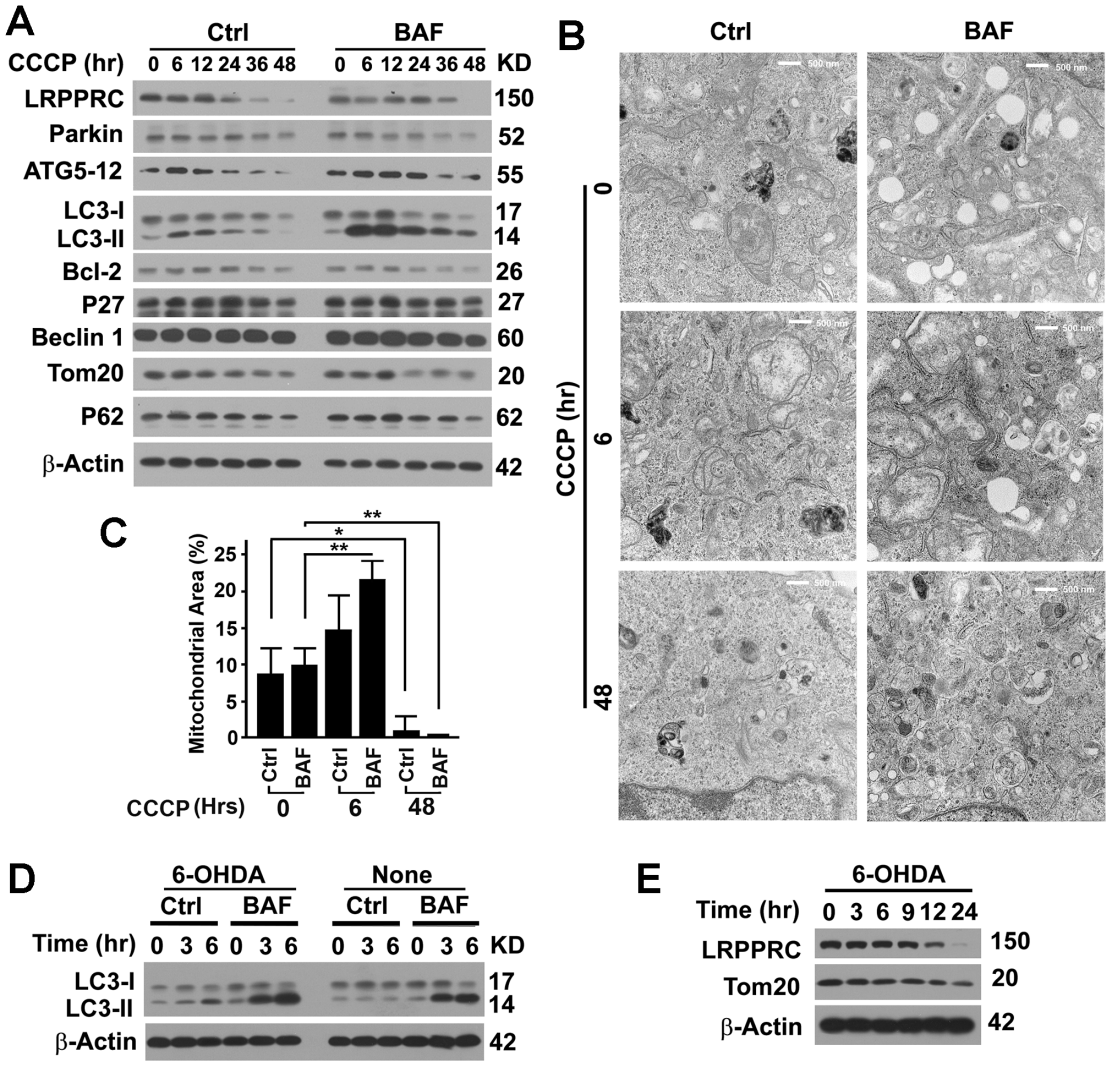
Long-term mitophagy stress results in depletion of LRPPRC and mitochondria and impairment of autophagy flux. (**A**) Immunoblot analysis of lysates from 293T cells treated with 10 µM carbonyl cyanide 3-chlorophenylhydrazone (CCCP) for different lengths of time (hrs) in the absence (Ctrl) or presence of lysosomal inhibitor Bafilomycin A1 (BAF, 10 µM) for 6 hrs before harvest. No Bafilomycin A1 was added in two treatments at time zero. (**B**) Representative TEM imaging of cells as treated in panel (**A**). No Bafilomycin A1 was added in two treatments at time zero. Bar = 500 nm. (**C**) Percentages of area occupied by mitochondria in the TEM images. Data were the average and standard deviation of at least three repeats and the differences are compared based on Student T-test. *, p value ≤0.05. **, p value ≤0.01. (**D,E**) Immunoblot analyses of lysates from 293T cells untreated (None) or treated with 100 µM 6-hydroxydopamine (6-OHDA) for different time (hrs) in the absence or presence of Bafilomycin A1 for 0–6 hrs (**D**) or 6 hrs before harvest (**E**).

LRPPRC suppresses the initiation of basal levels of autophagy and mitophagy via enhancing the stability of Bcl-2 protein [21]. Since LRPPRC is a mitochondrion-associated protein, we were interested to investigate its role in mitophagy, autophagy for turnover of mitochondria. As widely done in literatures [29], we used carbonyl cyanide 3-chlorophenylhydrazone (CCCP), a chemical causing uncoupling of mitochondrial potential, to induce mitophagy. We found that short-term CCCP treatment (20 µM for 6–12 hrs) did not change the levels of LRPPRC and Parkin but increased levels of ATG5-ATG12 conjugates, LC3-II and protein aggresomal marker P62 in 293T cells treated with lysosomal inhibitor Bafilomycin A1, suggesting that autophagy flux was elevated (Figure 1A). More swollen mitochondria were observed in cells that received CCCP treatment for 6 hrs than in untreated cells in the absence of Bafilomycin A1 and much higher mitochondria mass as reflected by the Tom20 levels remained in cells received CCCP treatment for 12 hrs than in untreated cells in the presence of Bafilomycin A1 (Figure 1A–C), suggesting that initial induction of mitophagy with CCCP may activate mitophagy. Since inhibition of lysosomal activity with Bafilomycin A1 was confirmed to have no impact on LRPPRC levels,[21] it is the long-term CCCP treatment that eventually led to depletion of LRPPRC and Bcl-2 but did not alter levels of Beclin 1 and P27 levels (Figure 1A), supposing to cause autophagy activation as we previously reported [16], [21]. However, the levels of ATG5-ATG12 conjugates were gradually reduced with prolonged period of CCCP treatment (Figure 1A). Activation of autophagy mediated by LRPPRC depletion occurs prior to ATG5-ATG12-mediated LC3-I to LC3-II conversion [21]. Similar to the situation as shown in the LRPPRC and ATG5 double knockdown experiments [21], LC3-II and P62 levels decreased in the absence or presence of Bafilomycin A1 48 hrs after CCCP exposure (Figure 1A), indicating an impairment of autophagy flux. Long-term CCCP treatment eventually led to depletion of mitochondrial mass as indicated by Tom20 levels in immunoblot and mitochondria-occupied area under electron microscopy, and mitophagy initiator Parkin (Figure 1A–C). Therefore, CCCP treatment initially led to activation of autophagy machinery and long-term CCCP insults led to depletion of mitochondria and the associated LRPPRC, and eventually caused impairment of autophagy flux.

Similarly, we treated 293T cells with 6-hydroxydopamine (6-OHDA), an oxidative toxin to generate experimental models of Parkinson's disease that is non-enzymatically oxidized to produce reactive oxygen species such as hydrogen peroxide to induce mitophagy [30]. We found that 6-OHDA behaved similar to CCCP but with accelerated pace, activated autophagy flux at early stage (Figure 1D) and resulted in reduction of Tom20-indictaed mitochondria mass and the mitochondrion-associated LRPPRC at late stage (Figure 1E). The fact that two drugs inducing mitophagy in different mechanisms have the same impact on LRPPRC levels and activities of autophagy and mitophagy suggests that LRPPRC plays a general role in mitophagy.

### LRPPRC interacts with mitophagy stress-induced mitochondrion-translocated mitophagy initiator Parkin

**Figure 2:**
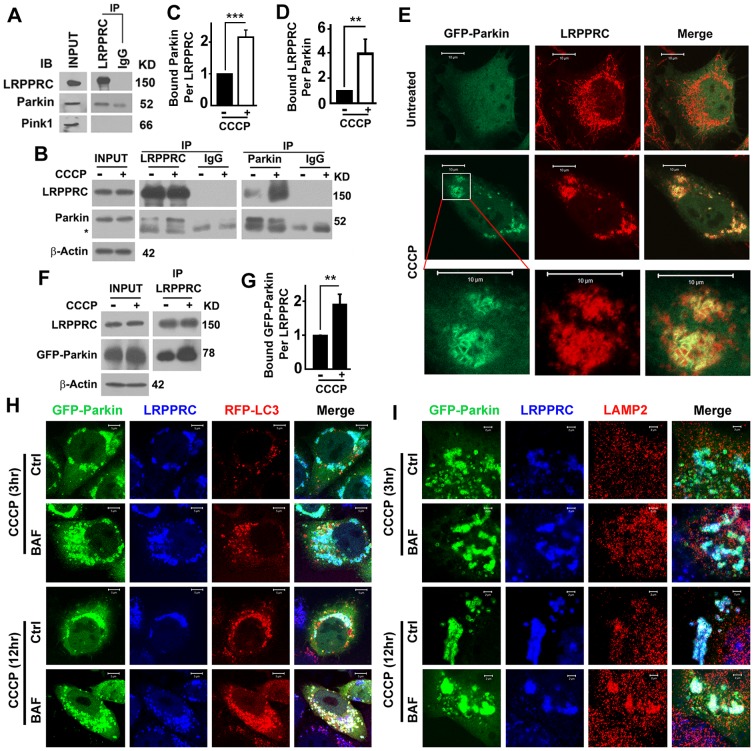
LRPPRC interacts with mitophagy stress-induced mitochondrion-translocated mitophagy initiator Parkin. (**A**) Immunoblot analyses of interaction between LRPPRC and Parkin or Pink 1. Same amount of 293T cell lysates were used to perform immunoprecipitation with same amount of anti-LRPPRC antibody or mouse IgG control under identical procedure. (**B**–**D**) Coimmunoprecipitation analyses of LRPPRC-Parkin interaction under mitophagy stress. Lysates with equal amount of total proteins prepared from 293T cells untreated or similarly treated with 10 µM CCCP for 2.5 hrs were immunoprecipitated with anti-LRPPRC or Parkin antibody (**B**). IgG served as control antibody. The relative amounts of Parkin bound on LRPPRC (**C**) or LRPPRC bound on Parkin (**D**) were quantified against the precipitated amounts of LRPPRC (**C**) or Parkin (**D**). The level in the absence of CCCP is set to 1. **, p value ≤0.01. (**E**) A fluorescent imaging analysis showing the colocalization of LRPPRC with GFP-Parkin. CCCP, HeLa cells transiently expressing GFP-Parkin for 48 hrs were treated with 10 µM CCCP for 2.5 hrs before fixation. Bottom panel is the amplification of the square in the middle panel. Bar = 10 µm. (**F,G**) Coimmunoprecipitation analyses of LRPPRC-Parkin interaction in HeLa cells overexpressing GFP-Parkin untreated or similarly treated with CCCP as shown in (**E**). GFP-Parkin is coimmunoprecipitated with anti-LRPPRC antibody (**F**), and the relative amounts of GFP-Parkin bound on LRPPRC are quantified against the precipitated amounts of LRPPRC (**G**). The level in the absence of CCCP is set to 1. **, p value ≤0.01. (**H,I**) Fluorescent imaging analyses showing the colocalization of GFP-Parkin and LRPPRC with RFP-LC3 punctate foci (**H**) or LAMP2-labelled lysosomes (**I**) 3 or 12 hrs after exposure to CCCP in the absence (Ctrl) or presence of Bafilomycin A1 (BAF). Bar = 5 µm in (**H**) and 2 µm in (**I**).

Parkin and Pink1 were previously suggested to interact with LRPPRC as detected by Mass Spectrometry [23], [24], [31]. To understand the role of LRPPRC in mitophagy, we tested the interaction of LRPPRC with Parkin or Pink1. Co-immunoprecipitation of endogenous proteins revealed that LRPPRC interacted with Parkin but not with Pink 1 (Figure 2A). Upon CCCP treatment for 3 hrs to induce mitophagy in 293T cells, more endogenous Parkin proteins were precipitated with similar amount of immunoprecipitated LRPPRC, or more endogenous LRPPRC proteins were precipitated with less immunoprecipitated Parkin (Figure 2B–D). When HeLa cells transiently expressing GFP-Parkin were induced to commit mitophagy with CCCP for 3 hrs, the diffusing GFP-Parkin translocated to and colocalized with the mitochondrion-associated LRPPRC (Figure 2E). The short-term CCCP treatment did not change the levels of LRPPRC but dramatically increased the amount of LRPPRC-bound GFP-Parkin (Figure 2F–G). Thus, Parkin translocates to mitochondrion to induce rupture of outer mitochondrial membrane [32] and bind with the exposed inner mitochondrial membrane-associated LRPPRC under mitophagy stress.

### Both LRPPRC and Parkin are co-localized with LC3 punctate foci and LAMP2-labelled lysosomes under mitophagy stress

When HeLa cells stably expressing RFP-LC3 and transiently expressing GFP-Parkin were exposure to CCCP for 3 hrs, we found that both LRPPRC and Parkin were not obviously colocalized with RFP-LC3 punctate foci even in the presence of Bafilomycin A1 (Figure 2H), suggesting the damaged parkin and LRPPRC-associated mitochondria were aggregated but not packaged into LC3-associated autophagosomes yet. After 12 hrs exposure to CCCP, colocalization of LRPPRC and Parkin-associated mitochondria with RFP-LC3 punctate foci were detected in the absence but became obvious in the presence of Bafilomycin A1 (Figure 2H, white in merge), indicating the damaged parkin and LRPPRC-associated mitochondria were packaged into LC3-associated autophagosomes for effective turnover. This was further confirmed by the colocalization of Parkin and LRPPRC-associated mitochondria with LAMP2-labelled lysosomes (Figure 2I).

### Under mitophagy stress, translocation of Parkin to LRPPRC-associated mitochondria induces mitochondrial aggregation and consequently degradation of LRPPRC, Parkin and mitochondria

**Figure 3:**
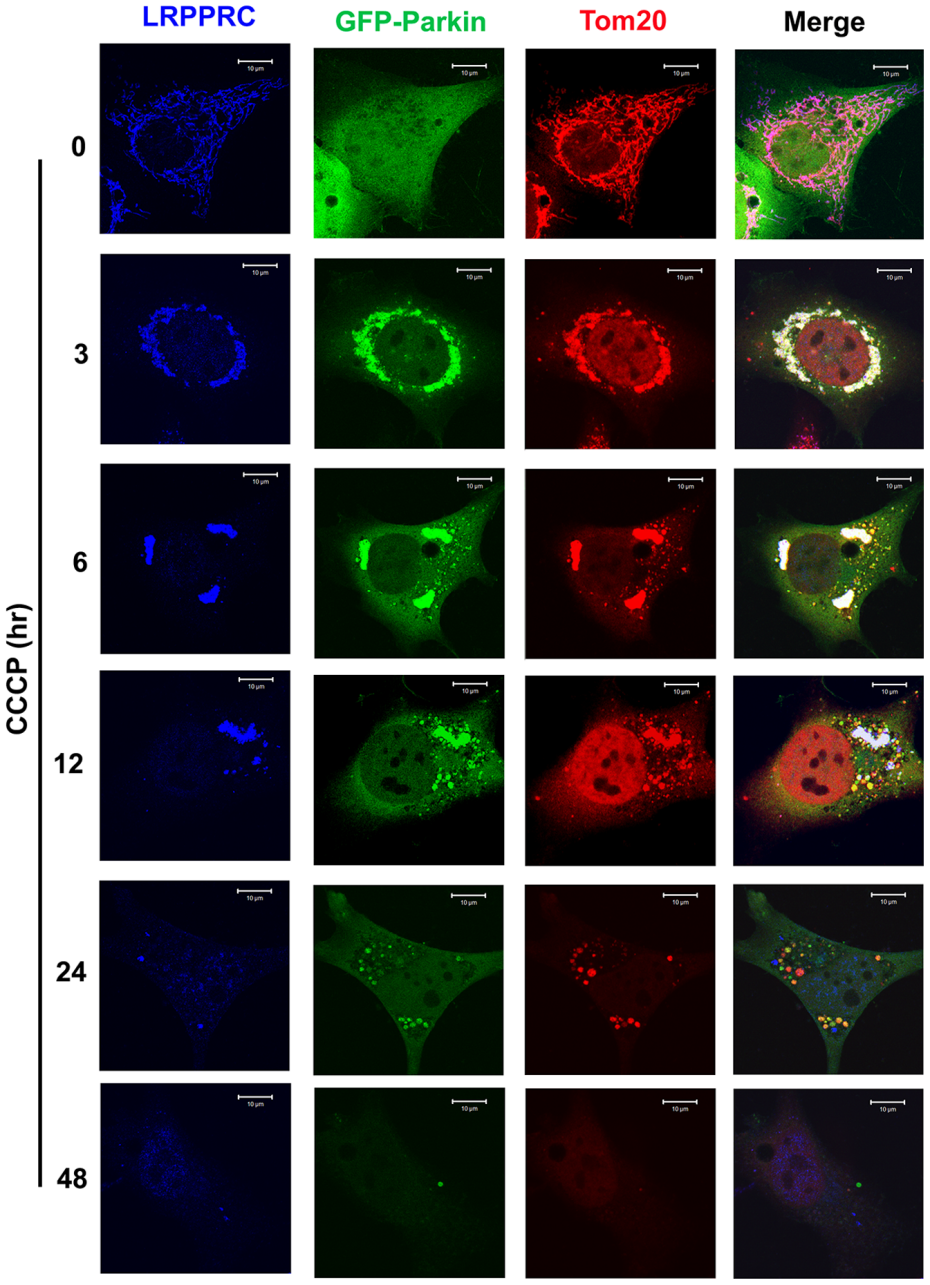
Colocalization among LRPPRC, Parkin and Tom20-indicated mitochondria at different times under mitophagy stress. HeLa cells transiently expressing GFP-Parkin treated with 10 µM CCCP for different times were fixed and stained with antibodies against LRPPRC (blue) and Tom20 (red). Mitochondrial aggregates carrying both LRPPRC and Parkin signals were shown as white in the Merge panels. Bar = 10 µm.

To understand mutual impact of LRPPRC and Parkin during mitophagy process, we examined their distribution on mitochondria at different times after exposure to CCCP (Figure 3). LRPPRC associated with Tom20-labelled mitochondria and overexpressed GFP-Parkin diffused in cytosol of untreated normal HeLa cells. GFP-Parkin translocated to LRPPRC-associated mitochondria and induced mitochondrial aggregation at 3 hrs after CCCP treatment. Mitochondrial aggregates gradually disappeared with prolonged periods of CCCP treatment from 6 to 24 hrs. A lot of GFP-Parkin-associated and Tom20-labelled mitochondrial fragments containing no LRPPRC (yellow foci in the merge panel) existed at 6 to 24 hrs after CCCP exposure. Finally, mitochondria and the associated LRPPRC and Parkin were depleted in cells after 48 hrs CCCP treatment.

**Figure 4:**
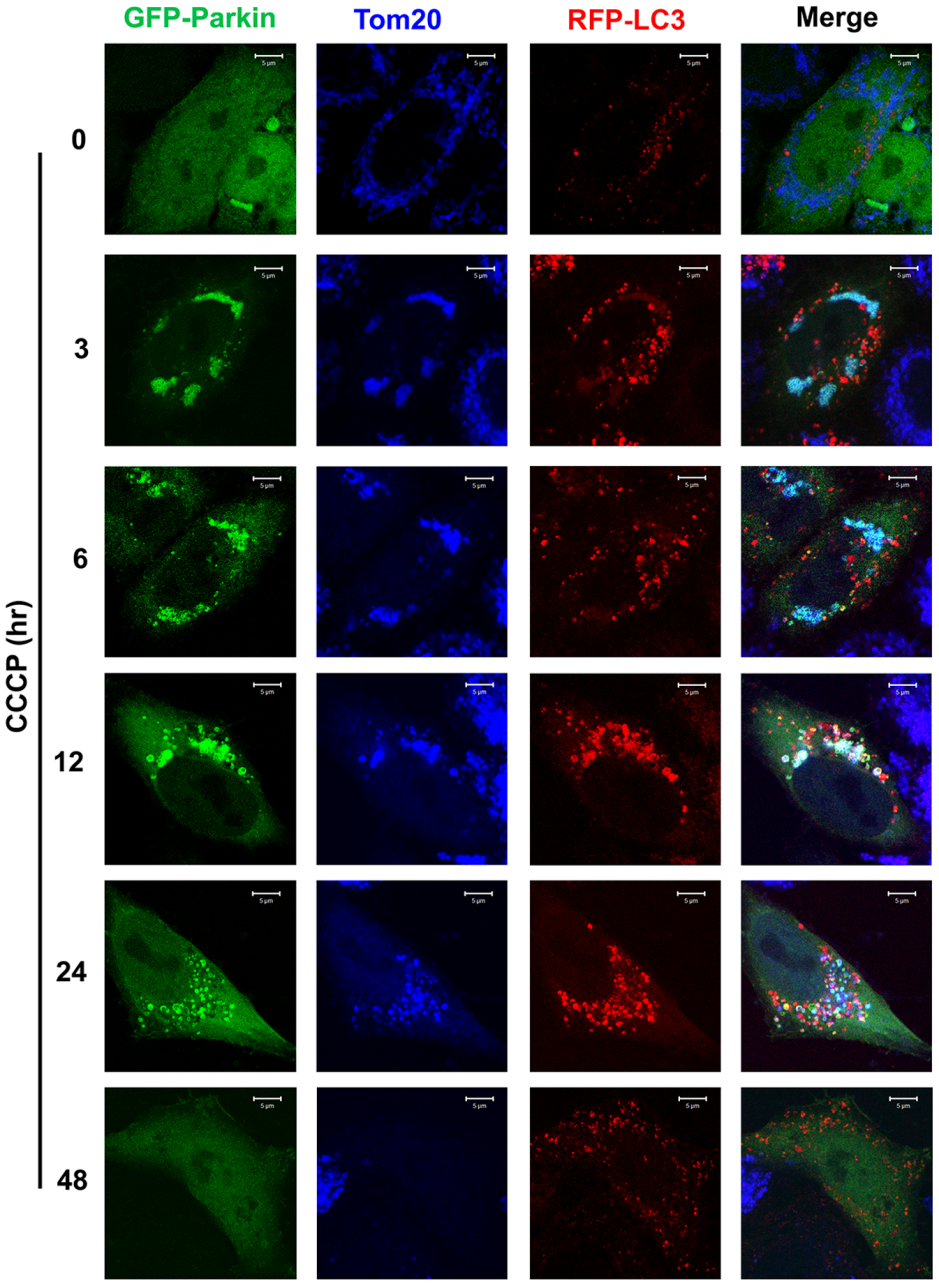
Colocalization among Parkin, Tom20-indicated mitochondria and RFP-LC3-labelled autophagosomes at different times under mitophagy stress. HeLa cells stably expressing RFP-LC3 transiently expressed GFP-Parkin treated with 10 µM CCCP for different times were fixed and stained with antibodies against Tom20 (blue). Mitochondrial aggregates associating with Parkin signals were shown as cyan in the Merge panels. Bar = 5 µm.

**Figure 5:**
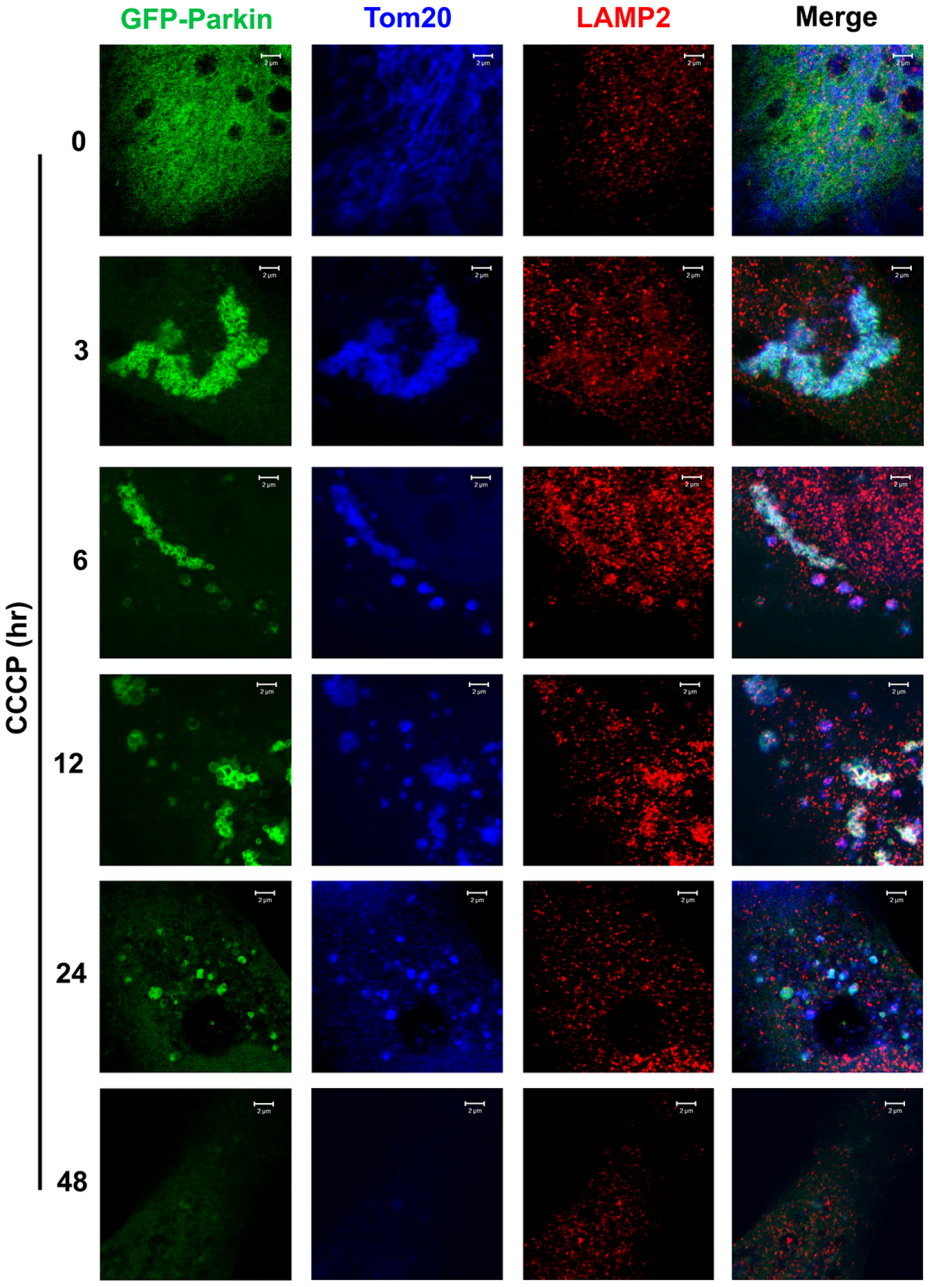
Colocalization among Parkin, Tom20-indicated mitochondria and LAMP2-labelled lysosomes at different times under mitophagy stress. HeLa cells transiently expressing GFP-Parkin treated with 10 µM CCCP for different times were fixed and stained with antibodies against LAMP2 (red) and Tom20 (blue). Lysosome-contained Parkin-associated mitochondrial aggregates were shown as white in the Merge panels. All experiments were carried out in the absence of lysosomal inhibitor. Bar = 2 µm.

Similar to as shown in Figure 2H, most of mitochondria were associated with Parkin and Parkin-associated mitochondria were not found to be colocalized with RFP-LC3 punctate foci or LAMP2-labelled lysosomes in in early time after CCCP exposure in the absence of Bafilomycin A1 (Figure 4,5). Those mitochondria were effectively packaged into autophagosomes but not effectively degraded through lysosomes so that we were able to observe some mitochondria-containing autophagosomes accumulated at 12 hrs after exposure (Figure 5). Those mitochondria free of LRPPRC but associating with Parkin were not colocalized with RFP-LC3 punctate foci or LAMP2-labelled lysosomes at 24 hrs after exposure (Figure 4,5), indicating a positive role of LRPPRC for mitochondria to be packaged into autophagosomes. Finally, mitochondria disappeared 48 hrs after exposure (Figure 4,5).

### LRPPRC and Parkin mutually regulate stability of each other

**Figure 6:**
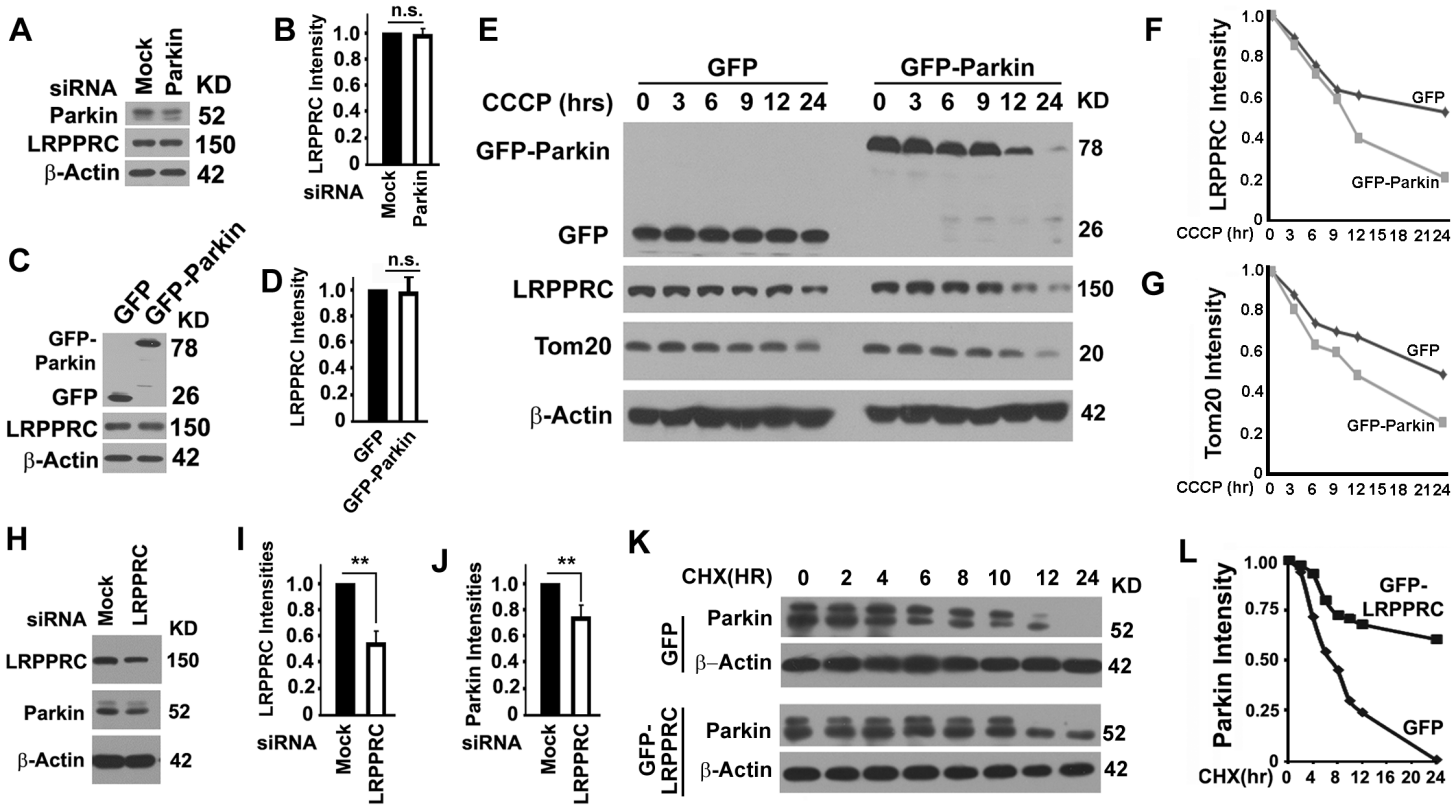
LRPPRC controls the stability of Parkin. (**A**–**D**) Immunoblot analyses showing the impact of different levels of Parkin on the levels of LRPPRC. Equal amount of cell lysates from 293T cells treated with random (Mock) or Parkin-specific siRNA (**A**) or HeLa cells overexpressing GFP or GFP-Parkin (**C**) were analyzed by immunoblot and LRPPRC levels under different conditions were quantified (B,D). n.s., not significant. (**E**) Immunoblot analyses showing the impact of different levels of Parkin on the levels of LRPPRC under mitophagy stress. Equal amount of cell lysates from HeLa cells overexpressing GFP or GFP-Parkin treated with CCCP for different times were analyzed by immunoblot. (**F,G**) Plots of relative intensities of LRPPRC (**E**) or Tom20 (**F**) in HeLa cells treated as in (**E**). The intensities in samples at time zero were set to 1. (**H**) An immunoblot analysis showing that suppression of LRPPRC resulted in degradation of Parkin. 293T cells were treated with Mock or LRPPRC siRNA for 72 hrs. Equal amount of lysates were analyzed. (**I,J**) Plots of relative intensities of LRPPRC (**I**) or Parkin (**J**) in 293T cells treated with Mock or LRPPRC siRNA. The intensities in samples treated with MOCK siRNA were set to 1. Data were the average and standard deviation of at least three repeats and the differences were compared based on paired T-test. *, p value ≤0.05; ***, p value ≤0.0001. (**K**) Immunoblot analyses showing that overexpression of LRPPRC enhanced the stability of Parkin. COS7 cells were transiently transfected with plasmids carry only GFP or GFP-LRPPRC for 24 hrs, detached and distributed equally to 8 wells. Each well was treated with cycloheximide (CHX) for different times (hrs). Equal amount of lysates as indicated by total protein concentration and β-actin control were analyzed. (**L**) Plots of relative intensities of Parkin in COS7 cells treated as in (**K**). The intensities in samples at time zero were set to 1.

To understand the relation between LRPPRC and Parkin, we first changed the levels of Parkin by transfection with a Parkin-specific siRNA in 293T cells or a plasmid expressing GFP-Parkin in HeLa cells. Either suppression or overexpression of Parkin caused no change in LRPPRC levels (Figure 6A–D), suggesting Parkin did not have impact on the levels of LRPPRC under normal condition. When HeLa cells overexpressing Parkin were treated with CCCP, LRPPRC was destabilized and mitophagy was enhanced because of decrease of Tom20 levels (Figure 6E–G). Suppression of LRPPRC with LRPPRC-specific siRNA in 293T cells led to significant decrease of both LRPPRC and Parkin levels (Figure 6H–J). Overexpression of GFP-LRPPRC increased the half-life of Parkin from about 6 hrs to more than 24 hrs and dramatically enhanced the stability of LRPPRC (Figure 6K,L). Therefore, LRPPRC maintained the stability of Parkin and mitochondrial translocation of Parkin under mitophagy stress led to subsequent degradation of both LRPPRC and Parkin.

### LRPPRC suppresses autophagy/mitophagy through controlling the Parkin stability

**Figure 7:**
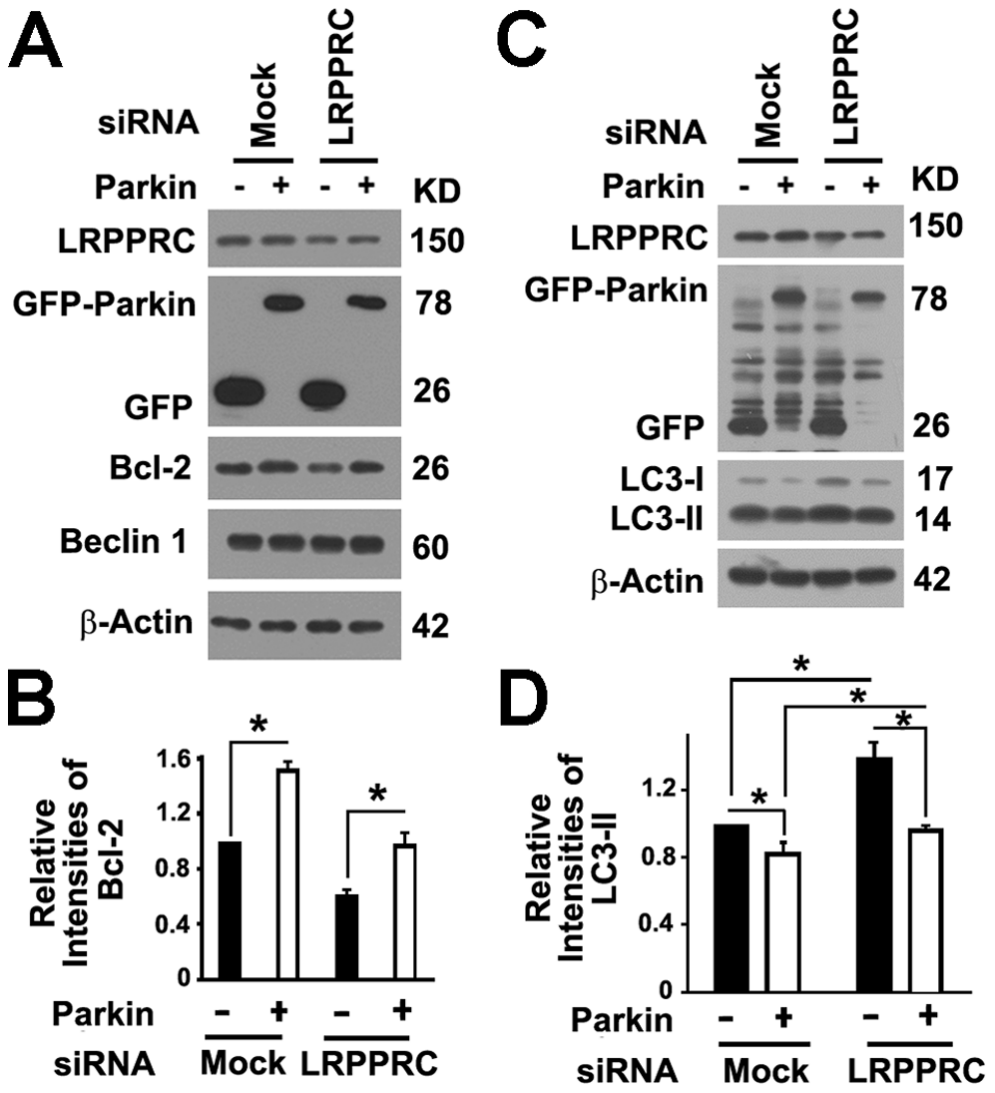
LRPPRC maintains levels of Bcl-2 and suppresses basal levels of autophagy through Parkin. (**A**) Immunoblot analyses of Bcl-2 levels in 293T cells overexpressing Parkin in the absence or presence of LRPPRC siRNA. (**B**) Plots of relative intensities of Bcl-2 as shown in (**A**). The intensities in samples overexpressing GFP were set to 1. Data were the average and standard deviation of at least three repeats and the differences were compared based on paired T-test. *, p value ≤0.05. (**C**) Immunoblot analyses of LC3-II levels in 293T cells treated with either random or LRPPRC-specific siRNAs and/or overexpressing GFP or GFP-Parkin in the presence of Bafilomycin A1. (**D**) Plots of relative intensities of LC3-II as shown in (**C**). The intensities in samples treated with random siRNA and overexpressing GFP were set to 1. Data were the average and standard deviation of at least three repeats and the differences were compared based on paired T-test. *, p value ≤0.05.

As an E3 ubiquitin ligase, Parkin binds with Bcl-2 and mono-ubiquitinates it to enhance its stability [33]. As predicated, overexpression of Parkin led to increases in Bcl-2 levels in cells containing either normal or suppressed levels of LRPPRC and suppression of LRPPRC led to destabilization of Parkin and further decrease of Bcl-2 levels (Figure 7A,B). Consistent with our previous report [21], suppression of LRPPRC led to activation of basal levels of autophagy, and overexpression of Parkin that caused elevation of Bcl-2 levels led to inhibition of basal levels of autophagy as indicated by the reduced levels of LC3-II in the presence of Bafilomycin A1 (Figure 7C,D).

**Figure 8:**
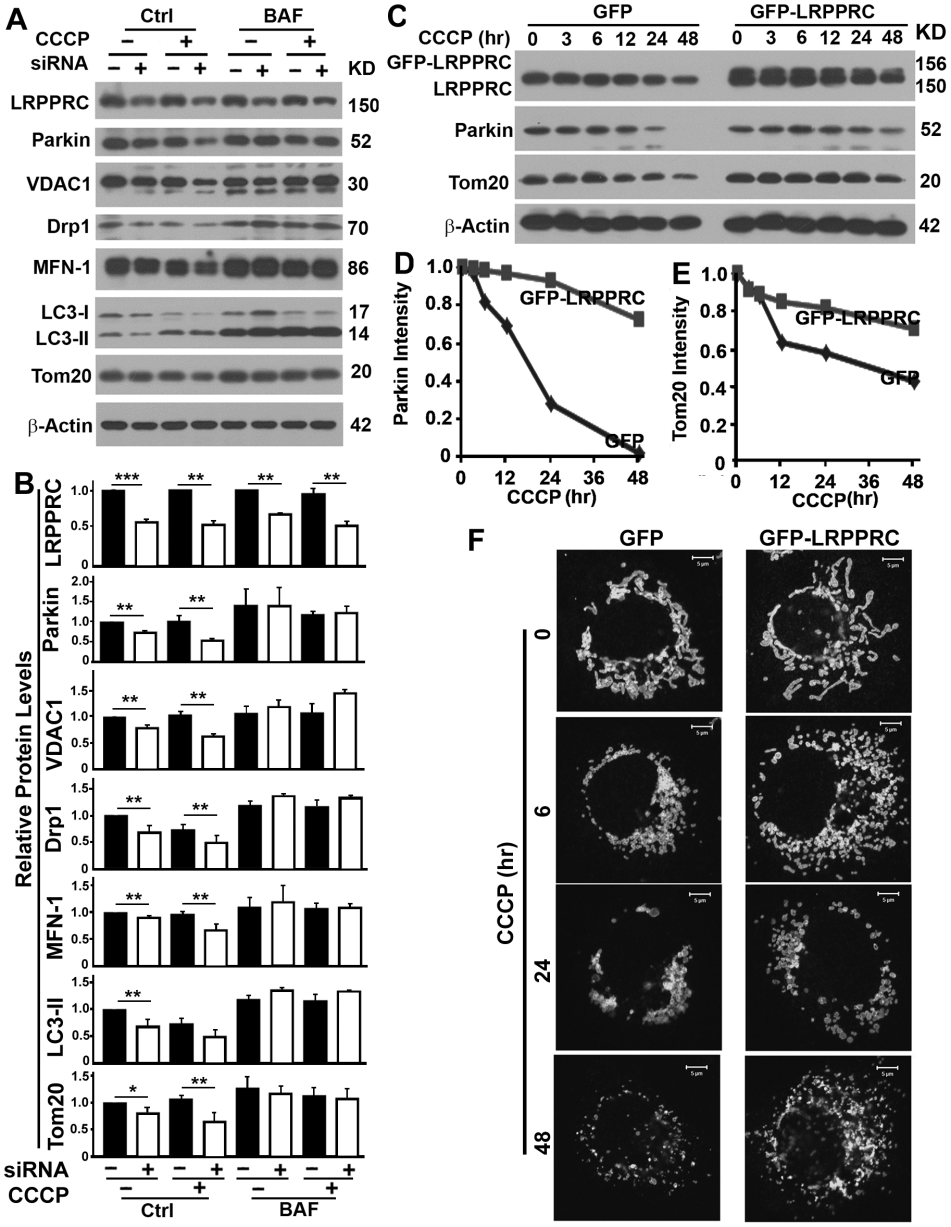
LRPPRC suppresses mitophagy through controlling the Parkin stability. (**A,B**) Immunoblot analyses of impact of LRPPRC on Parkin, Parkin substrate VDAC1, Drp1 and Mitofusion, and autophagy/mitophagy in 293T cells. Lysates with equal amount of total proteins were prepared from cells treated with random (Mock) or LRPPRC-specific siRNA and induced mitophagy with 0 or 10 µM CCCP in the absence or presence of Bafilomysin A1. Same amounts of cell lysates were subjected to immunoblot analyses (A) and the relative protein levels to those of β-Actin were plotted (B). **, p value ≤0.01. (**C**) Immunoblot analyses of impact of LRPPRC on Parkin and mitophagy in COS7 cells overexpressing GFP or GFP-LRPPRC treated with 10 µM CCCP for different times (hrs). (**D,E**) Plots of relative intensity of Parkin (**D**) or Tom20 (**E**) for immunoblots shown in (**C**). The intensities in samples collected at 0 hr were adjusted based on β-Actin intensity and set to 1. (**F**) Immunostaining analysis of mitochondrial mass in COS7 cells similarly treated as in (**C**) showing the intensities of Tom20-labelled mitochondrial mass. All images were captured under identical protocol of staining and imaging settings.

Suppression of LRPPRC caused degradation of Parkin and further treatment with CCCP resulted in faster degradation of Parkin (Figure 8A,B). Mitochondria have been suggested to be docked into autophagosomes via the interaction of ubiquitinated membrane proteins with LC3-II-interactive substrate receptor P62. Mitochondria-associated protein Mitofusin 1, Drp1 and VDAC1 have been confirmed as the substrates of Parkin E3 ligase and claimed as the P62-interactive membrane proteins but such claim is still in disputation [7]. When cells with LRPPRC suppressed were exposed to mitophagy inducer CCCP, activated E3 ligase activity of Parkin led to decreases in levels of VDAC1, Drp1 and Mitofusin 1 caused by faster turnover through autophagy (Figure 8A,B).

As we previously reported [21], LRPPRC suppression led to autophagy activation as indicated by LC3-II levels in the presence of Bafilomycin A1 (Figure 8A,B). Activated autophagy reduced the levels of Tom20 and enhanced the degradation of TOM20-labelled mitochondria (Figure 8A,B). Prolonged periods of CCCP treatment led to gradual degradation of Parkin and Tom20-labelled mitochondria and overexpression of LRPPRC maintained the stability of Parkin and prevented the degradation of Tom20-labelled mitochondria (Figure 8C–F).

## Discussion

The anti-apoptotic proteins of Bcl-2 family exhibit opposite impact on autophagy initiation. Our previous report shows that LRPPRC controls the stability of Bcl-2 to suppress basal levels of autophagy mainly through the Beclin 1-depdendent PI3K-AKT-mTOR pathway [21]. Here we demonstrate that LRPPRC interacts with Parkin and maintains its stability so that the Parkin substrates including Bcl-2 and Parkin itself are stabilized. Thus, LRPPRC protects mitochondria from autophagy degradation. Under mitophagy stress, Parkin translocates to mitochondrion to induce rupture of outer mitochondrial membrane [32] and bind with LRPPRC. Then, LRPPRC, Parkin and other substrates of Parkin may be ubiquitinated by Parkin E3 ligase and recognized by autophagy machinery and guide mitochondria to be degraded through mitophagy.

Parkin is selectively recruited to dysfunctional mitochondria with low membrane potential in mammalian cells. After recruitment, Parkin mediates the engulfment of mitochondria by autophagosomes and the selective elimination of impaired mitochondria [34]. Mitofusin 1 [35], Drp1 [36] and VDAC1 [6] were reported to be substrates of Parkin while LRPPRC is also listed as Parkin substrates in the associated online supplementary although the exact mechanism is still in investigation [37]. Ubiquitinated VDAC1 and Drp1 will cause their associated mitochondria to be brought into autophagosomes and autolysosomes for degradation. Since a significant portion of Bcl-2 is associated with mitochondria and Parkin-mono-ubiquitinated Bcl-2 is more stable, suppression of LRPPRC leads to decreases in levels of Parkin and Bcl-2 and activation of basal autophagy as we previous reported [21]. Interestingly, Parkin itself is the substrate of its ligase activity. After auto-ubiquitination, Parkin gradually becomes depleted along Bcl-2 and ATG5-ATG12 conjugate in cells under long-term mitophagy stress.

The drug-induced mitophagy stress is an artificially introduced pathological condition. Under normal physiological condition, it is unlikely that all of mitochondria in cells are simultaneously damaged. The drug-induced mitochondrial damages are so massive that autophagy/mitophagy machinery is incapable of handing so many damaged mitochondria immediately. This is possibly the reason that we observed a large amount of mitochondria aggregates accumulated in the first 12 hrs after exposure to mitophagy inducer. These mitochondrial aggregates then become fragmented mitochondria to be engulfed in autophagosomes and further autolysosomes for degradation.

Parkinson's disease results from the death of dopamine-containing cells in the substantia nigra region of the midbrain. Several mutations in specific genes such as Parkin have been identified in a few individuals with familial form or autosomal recessive juvenile Parkinson's disease [38]. Mitochondrial dysfunction and oxidative stress have long been implicated as the general pathophysiologic mechanisms underlying Parkinson's disease [39]. Impairment of autophagy and mitophagy processes may be the determining force in the majority of patients to develop Parkinson's disease [39].

Interestingly, the same group of proteins involved in juvenile Parkinson's disease also plays important roles in tumorigenesis although the somatic mutations of Parkin identified are homozygous in Parkinson's disease and heterozygous in cancers [40]. If the autophagic process is blocked before autophagosomal formation, the fragmented mitochondria will release cytochrome *c* and other molecules to induce apoptosis that is usually associated with diverse forms of aggregation and perinuclear clustering of the dysfunctional mitochondria [41], [42]. If either the process is blocked before the autolysosomal formation or autophagosomes are not degraded efficiently, the accumulated mitochondria may become damaged by their own production of superoxide and start to leak electrons and lose their membrane potentials, and even further induce robust oxidative stress [43]. High levels of oxidative stress are lethal in post-mitotic neuronal cells in Parkinson's disease, while sub-lethal levels of oxidative stress not only induces DNA double-strand breaks but also weakens mitotic checkpoint function so that cells carrying damaged genomes can escape mitotic checkpoint to enter next rounds of mitosis to further destabilize the genomes and result in tumorigenesis [16]–[18]. High levels of LRPPRC maintain Bcl-2 levels, block mitophagy and prevent mitochondria from autophagy degradation. It has been known that overexpression of members of the Bcl-2 family of pro-survival proteins is commonly associated with unfavorable pathogenesis in cancer [44]. Specifically, high levels of Bcl-2 protein are detected in androgen-independent tumors in advanced stages of the pathology [45]. It is well known that most tumor cells need more energy than their normal mature counterparts [46]. Prostate cancer, like other cancers, demonstrates abnormal mitochondria activity [47], [48]. Therefore, patients at late stage of prostate adenocarcinomas exhibit higher levels of LRPPRC than those at early stage of the disease [19].

Since LRPPRC-Parkin interaction may play important roles in two seemingly contradicting events of uncontrolled cell growth in cancers and cell death and neuron degeneration in Parkinson's disease, manipulating the interaction provides a new opportunity to target both diseases. Characterization of exact interactive domains in both LRPPRC and Parkin proteins may help to develop drugs to precisely regulate the interaction to differentiate their specific roles from those played by other interactive proteins.
